# Exploring the Influence of LGBTQ+ and Religious Attitudes in Sexual Health Education for College Students With Intellectual and Developmental Disabilities

**DOI:** 10.1111/jar.70221

**Published:** 2026-03-31

**Authors:** Kristin J. Lurie, Rebekkah L. Gross, Raeshann Berry, Lori B. Vincent, Brittany E. Hayes, Kerri A. Wolfer

**Affiliations:** ^1^ School of Criminal Justice University of Cincinnati Cincinnati Ohio USA; ^2^ School of Human Services University of Cincinnati Cincinnati Ohio USA; ^3^ School of Education University of Cincinnati Cincinnati Ohio USA

**Keywords:** attitudes, intellectual and developmental disabilities, LGBTQ+, religion, sexual health education

## Abstract

**Background:**

Comprehensive sexual health education is a human right routinely denied to people with intellectual and developmental disabilities. Although queerness and religion are critical parts of comprehensive sexual health education, they are rarely addressed in the literature.

**Methods:**

Fifteen college students with intellectual and developmental disabilities enrolled in a programme that includes a sexual health education course participated in semi‐structured interviews about the class. Responses related to attitudes about religion, gender identity and sexual orientation were thematically analysed.

**Results:**

Participants expressed varied views about religion and queerness influenced by factors including the desexualisation stereotype (the widespread idea that disabled people should not be sexually active), access to formal and informal sexual health education and restrictive religious teachings.

**Conclusions:**

The diversity in perspectives shown here is an important consideration for the development of sexual health education programmes for people with intellectual and developmental disabilities in educational and community‐based settings.

## Summary

- College students with intellectual and developmental disabilities have a variety of views about how religion and LGBTQ+ identity are related to sexuality.
- In this study, participants explained how the sexual health education they had gotten before from school and outside sources affected their thoughts about religion, LGBTQ+ identity and sexuality.
- This study adds to our knowledge about how to deliver sexual health education to young adults with intellectual and developmental disabilities and will help make sexual health education classes better in the future by understanding the diverse beliefs that students have. This, in turn, makes the classroom a place where everyone feels free to discuss their ideas.

## 1 Introduction

Several international human rights organisations include access to comprehensive sexual health education in their standards for children's rights (Miller et al. 2015). In the United States, sexual health education curricula often fail to meet these standards, especially for people with intellectual and developmental disabilities.^1^ Much of the sexual health education that people with intellectual and developmental disabilities receive focuses on preventing victimisation or promoting abstinence (Onstot 2019; Travers et al. 2014). These curricula fall short of the standards set by human rights organisations on comprehensive sexual health education, which should include information on anatomy and puberty, consent and bodily autonomy, relationships and parenting, pregnancy and contraception and sexually transmitted infections (Miller et al. 2015).

Self‐advocates with intellectual and developmental disabilities see this education as a priority because people with intellectual and developmental disabilities are at an elevated risk of experiencing sexual victimisation (Harrell 2021) or offending sexually, potentially due to a lack of education on consent and other sexual behaviours (Martinello 2015). Self‐advocates believe that explicit instruction on sexuality and sexual health would mitigate this risk by dispelling misinformation about sexual health, to which people with intellectual and developmental disabilities are particularly susceptible. However, people with intellectual and developmental disabilities rarely receive such instruction (Brown and McCann 2018). Several factors contribute to this gap in education, such as the enduring stigma that adults with intellectual and developmental disabilities are asexual and incapable of exploring their sexuality (Cuthbert 2017; Onstot 2019), insufficient preparation and support for teachers to effectively deliver sexual health education to this population, and parent and teacher discomfort with teaching about sexual health (Greene et al. 2024; Tamas et al. 2019).

Despite these barriers to sexual health education, people with intellectual and developmental disabilities are increasingly receiving comprehensive sexual health education at universities (Pratt 2025). The number of individuals with intellectual and developmental disabilities pursuing higher education has increased (Lee and Taylor 2022). Across the US, approximately 300 colleges and universities have instituted formal programmes to support the inclusion of undergraduate students with intellectual and developmental disabilities (Lee and Taylor 2022). These programmes, although diverse in design and execution, collectively empower thousands of students with intellectual and developmental disabilities to engage in college courses, dormitory life and social opportunities unique to college campuses (Kirkendall et al. 2009). The presence of these programmes represents a significant stride toward providing equal and inclusive educational opportunities for individuals with intellectual and developmental disabilities. However, it is essential to acknowledge that, based on interviews with professionals working with young adults with intellectual and developmental disabilities, considerable gaps in sexual health education for this community still exist (Crehan et al. 2023).

Comprehensive sexual health education for students with intellectual and developmental disabilities should include materials delivered in a queer‐ and disability‐inclusive manner, including queer content and resources created by queer individuals within the curricula (Tarasoff 2021). Important to this discussion is that disabled people are more likely to be LGBTQ+ than the general population (Leonard and Mann 2018), and gender nonconformity is particularly common among people with intellectual and developmental disabilities (Warrier et al. 2020). Researchers have identified a particularly strong connection between being autistic and identifying as a sexual minority (George and Stokes 2018; Rudolph et al. 2018). Some theorists and self‐advocates attribute this to disabled people already not fitting society's notion of ‘normal’, which may result in less pressure to conform to cisheteronormative expectations (Cuthbert 2017). Caregivers and teachers know that they have limited knowledge about appropriate sexual health education with adults with intellectual and developmental disabilities, especially those who identify as LGBTQ+ (Crehan et al. 2023). Due to this lack of knowledge and support, misinformation and stereotypes often guide LGBTQ+ sexual health education for adults with intellectual and developmental disabilities (Schmidt et al. 2021).

Religion can also play a significant role in an individual's view of sexuality and sexual behaviour. Thornton and Camburn (1989) found that the religious views of young adults typically resemble those of their community or caregivers. As many caregivers and educators of young adults with intellectual and developmental disabilities have religion‐based, conservative sexual views (Tamas et al. 2019), much of the informal education that would be received within this context tends to skew conservatively and neglect the sexual expression of the recipient (Santinele Martino 2022). This informal education often focuses on the victimisation and desexualisation of adults with intellectual and developmental disabilities (Travers et al. 2014). When caregivers' religious beliefs and the needs of people with intellectual and developmental disabilities are at odds, religious conflict may occur and may further limit the sexual health education received (Mintz 2018). While some is known about the knowledge and attitudes of caregivers and teachers with respect to LGBTQ+ identity and religion in the context of sexual health education (Crehan et al. 2023; Greene et al. 2024; Tamas et al. 2019; Turner et al. 2004), no prior studies have investigated the attitudes of people with intellectual and developmental disabilities themselves—in their own words.

This article seeks to begin to address gaps in existing knowledge by exploring the attitudes of college students with intellectual and developmental disabilities towards queerness and religion in the context of sexual health education. It will also expand the literature on how religion impacts views about sexual health education among students with intellectual and developmental disabilities. This study examined the following research questions:

1. What level of knowledge do college students with intellectual and developmental disabilities have about gender and sexual identity?
2. What is the influence of gender and sexual identity knowledge on initial attitudes about sexuality and sexual health?
3. What is the influence of religion on initial attitudes about sexuality and sexual health?

## 2 Methods

The current study is part of a larger longitudinal, mixed‐methods design that includes repeated qualitative, semi‐structured interviews and quantitative assessments to investigate changes in the sexual health knowledge and attitudes of students enrolled in a four‐year certificate programme for adults with mild‐to‐moderate intellectual and developmental disabilities at a Midwestern university. In the certificate programme, most students live on campus and attend university courses available to all students, as well as a specific curriculum and courses designed for the certificate programme. A critical part of the curriculum for students enrolled in the certificate programme is relationship and sexuality education, beginning with their first semester. This study was approved by the Institutional Review Board at the university of the authors. The procedures used in this study align with the principles of the Declaration of Helsinki. Details regarding the specific quantitative instruments used in the larger study and their psychometric properties are outside the scope of this paper.

### 2.1 Participants

Participant demographics are included in Table 1. All students were in their first or second year of the four‐year college certificate programme. Our sample was largely White and Christian.

**TABLE 1:** Demographic information for sample (*n* = 15).

Pseudonym	Gender	Class	Race	Diagnosis	Sexual identity	Religion^a^
Oscar	Male	1st year	White & American Indian/Alaska Native	Down syndrome; HOH; ID	Mostly heterosexual	Jewish
Brian	Male	1st year	American Indian/Alaska Native	—	Heterosexual	Christian
Hugh	Male	1st year	White	Chromosomal	Heterosexual	Christian
Brett	Male	1st year	White	ADHD; Chromosomal; HOH	Heterosexual	Christian
Jack	Male	2nd year	White	Autism	Bisexual	Christian
Dennis	Male	2nd year	White	Autism; ID	Bisexual	Christian
Marshall	Male	2nd year	White	Autism; Cerebral palsy; Deaf; ID	Heterosexual	Catholic
Harriet	Female	1st year	White	ADHD; Cerebral palsy; Epilepsy	Bisexual	Catholic
Angelina	Female	1st year	White	ADHD; Autism; ID	Heterosexual	Christian
Cecilia	Female	1st year	White	ID	Heterosexual	Christian
Leah	Female	1st year	White	ADHD; Autism; ID	Homosexual	No affiliation (Former Catholic)
Isabel	Female	1st year	Black/African American	—	—	Catholic
Pamela	Female	1st year	White	ADHD	—	Catholic
Serena	Female	2nd year	White	—	Heterosexual	Catholic
Lillian	Female	2nd year	White	Autism; ID	Heterosexual	—

*Note:* All names were changed to pseudonyms. Dashes indicate that the participant preferred not to answer. Abbreviations: ADHD, Attention‐deficit hyperactivity disorder; Chromosomal, Rare chromosomal condition; HOH, Hard of hearing; ID, Intellectual disability.

^a^ The survey listed ‘Christian’ without asking about denominations; however, it included ‘Catholic’ as a separate option.

### 2.2 Procedures

Before conducting interviews, informed consent was obtained from all study participants. For participants with a legal guardian, both the participant and their guardian gave informed consent. Participants received additional information about concepts relevant to the study in related courses before the interviews began to support their understanding of the questions that would be asked. Interviews included demographic information (e.g., gender identity, sexual orientation, and religious affiliation), questionnaires from a larger research study and a semi‐structured interview that included questions related to LGBTQ+ identity, religious identity and associated attitudes. The interviewers asked questions in a way that aligned with the instruction delivered to students and were prepared to answer questions that arose throughout the interview process. The interview setting was designed to accommodate the needs of students with intellectual and developmental disabilities. For example, the rooms had minimal furniture and décor, creating a low‐distraction environment and were in a familiar part of the building, which mitigated some discomfort, as did the private nature of the rooms. Fidget toys were provided to alleviate anxiety and signal to participants that self‐stimulatory behaviour would be accepted. The semi‐structured interviews included questions that elicited more in‐depth answers about interest, comfort and factors that potentially influence how they view sexuality and sexual health, such as prior formal and informal sexual health education. To protect the privacy of the participants, these data cannot be made publicly available.

### 2.3 Positionality

Our research team includes both researchers who identify as having a disability and who identify as nondisabled. One research team member identifies as autistic, which she would disclose to autistic participants to increase comfort during interviews and reduce the need for masking on both sides. Three research team members have lived experience with ADHD. Several of the researchers are family members of a person(s) with an intellectual or developmental disability. The research team includes researchers of various identities related to sexuality and religion. The sexual identities of team members include lesbian, pansexual and heterosexual. Religious identities of team members include Jewish, Catholic and nonreligious. All research team members have a passion for the rights and understanding of perspectives of people with intellectual and developmental disabilities and for increasing the autonomy and self‐determination of people related to their sexual identities. Additionally, the research team desires to address the sexual assault of people with intellectual and developmental disabilities through education and empowerment.

## 3 Analytic Strategy

This study presents both the quantitative demographic data and qualitative interview data gathered during the interviews. The qualitative data gathered during the interviews came either in direct response to the semi‐structured interview questions or as comments given at other points of the research process, often while answering a survey question. Both types of remarks were treated the same way: to provide appropriate context to the quote, the words of the interviewer or the content of the question may be included.

The qualitative data were coded in several stages, guided by the principles of thematic analysis to identify shared experiences, thoughts and feelings related to queerness, religion and sexual health among the participants (Braun and Clarke 2006). Specifically, thematic analysis was chosen to allow the data to speak for itself rather than being bound by a theoretical framework before analysing the participants' responses. First, Otter. AI was used to start the transcription process, followed by each interviewer checking the accuracy of and editing the transcriptions of the interviews they conducted. Second, two members of the research team read all transcripts twice—once to understand the experiences the participant described and again to identify quotes relevant to gender, sexuality and religion. Each interviewer noted such quotes and categorised them according to their interpretation of the quote's content. Third, the interviewers met multiple times to work on integrating the descriptions they gave to the quotes. When disagreements arose about the interpretation of certain quotes, the interviewers discussed the assumptions they made about the meaning of the quote, and the interviewer who performed the interview relayed any nonverbal cues to clarify the meaning of the participants' words. Throughout analysis, themes were constructed through iterative team discussion and reflexive consideration of how the researchers' disciplinary backgrounds, professional experiences and commitments to disability‐affirming and trauma‐informed practice shaped analytic decisions. Fourth, once the interviewers reached a consensus, they created an inductive coding scheme based on their notes. These discussions culminated in the formation of three themes.

## 4 Results

Three major themes were constructed from the data during the thematic analysis: *Attitudes on LGBTQ+ Identities*, *Religious Beliefs and Attitudes* and *Homophobia in Religious Contexts*. Each subtheme within these broader themes is defined in Table 2 and described in detail below.

**TABLE 2:** Emergent themes and definitions.

Theme	Definition
Attitudes on LGBTQ+ identities	
Level of confidence in knowledge	Knowledge or confidence in their own gender identity and/or sexual orientation. Knowledge about the social dynamics of gender and/or pronouns. Lack of knowledge of terms related to sexual orientation and/or gender identity.
Desexualisation	Experiences a restriction or denial of their sexual autonomy by others (e.g., parents, caregivers, teachers and staff). Emphasises how respondents are treated as if their sexuality/gender identity is not their own to control or express without permission.
Informal education and sentiments about sexuality and gender	Discusses the role of informal sources of education, including social media, television and family members. Respondents offer positive, negative, or neutral thoughts about gay, lesbian, trans and/or nonbinary individuals.
Religious beliefs & attitudes	
Religion as not influential (on sexual/gender attitudes)	Does not acknowledge an impact of religion on sexual and/or gender attitudes. Inability to express whether religion affects sexuality and/or gender attitudes.
Religion as influential (on sexual/gender attitudes)	Acknowledges some effect of religion on sexual and/or gender attitudes.
Sexual conservatism	Attitudes and behaviours regarding sexuality are shaped by religious beliefs and institutional teachings in a restrictive manner.
Homophobia in religious contexts	
Homophobia in religious institutions	Addresses how homophobic sentiments are taught in religious contexts and how they reconcile these teachings with their personal beliefs.

### 4.1 Major Theme 1: Attitudes on LGBTQ+ Identities

Eleven of the 15 students responded to and elaborated on questions about sexual orientation and gender identity. Each of the three subthemes is discussed below.

#### 4.1.1 Subtheme 1.1: Level of Confidence in Knowledge About Sexual Orientation and Gender Identity

Most participants expressed confidence in their understanding of sexual orientation and gender identity and could immediately identify their sexual orientation and gender identity during the interview. Many responses to the demographic questions about gender and sexual identity resembled the exchange between Leah [homosexual, no religious affiliation] and the interviewer below:

Interviewer The first question asks what gender you identify with.

Leah Female.

Interviewer Do you consider yourself to be transgender?

Leah No.

Interviewer Okay. The next question asks about your sexual orientation.

Leah Lesbian.

Some participants also discussed the social dynamics of gender identity and sexual orientation, demonstrating their knowledge of the topic. After the interviewer asked if he thought that it is important for people with disabilities to know about sexuality and sexual health, Brett [heterosexual, Christian] said,

Knowing what gender somebody is, and all that stuff, is very important. You don't want to misgender someone or something; like, you don't want to offend somebody, but for a person that has a disability, it's a lot harder for them to understand it as a topic, but it is a topic that does need to be talked about in the US, I feel like.

Three participants expressed that they did not know what the words used in the sexual orientation and gender identity questions meant. The phrase ‘sexual orientation’ was the most problematic. After being asked about her sexual orientation, Cecilia [heterosexual, Christian] asked, ‘What does that mean?’ A pause after the interviewer asked Isabel [no specified sexuality, Catholic] that question led to the following exchange:

Interviewer Do you know what that means?

Isabel Oh, like something inappropriate?

#### 4.1.2 Subtheme 1.2: Desexualisation

Three participants, all women, made comments about not having complete control over their sexual activity. When asked how she thinks her disability affects her sexuality, Isabel [no specified sexuality, Catholic] said, ‘I'd ask permission from my mom and dad to have sexuality when I get older’. Angelina [heterosexual, Christian] also expressed that she ‘would have to have permission to do it’ from her parents. Both Angelina and Isabel said they had never taken a class about sexual health before and expressed disgust at the idea of learning about it. In contrast, Leah [homosexual, no religious affiliation] mentioned that her sister had helped reinforce that she has control over her sexuality: ‘One of the adults said, “You guys should not date until you're a sophomore!” I told my sister about that, and she was pissed! She was like, “You're almost 21. You can date if you want”’.

#### 4.1.3 Subtheme 1.3: Informal Education and Sentiments About Sexuality and Gender

Two participants brought up the role of online sources of informal education about sexuality and gender during their interviews. When he was asked about his readiness to engage with his sexuality, Brett [heterosexual, Christian] replied:

That's one of my fears when I open up to having sex with a woman. Is she comfortable with it? You know, at any time, even after she gives you consent, she can turn right around, and flip of a switch, you could get arrested. I'm not saying that's gonna happen, but…

Brett mentioned that he had learned to fear such situations after ‘see[ing] all those videos’ online about gender and sexuality. Leah [homosexual, no religious affiliation] also attributed much of her knowledge to social media. She referenced ‘Instagram [and] TikTok’ as leading her to reject gendered stereotypes about clothing, saying, ‘I believe that clothes have no gender. If a guy wants to wear a dress, go ahead. If a girl wants to wear a suit, go ahead’.

Conversely, Isabel [no specified sexuality, Catholic], a participant who struggled to identify her sexual orientation, mentioned that her parents restricted her media access, saying that her parents ‘need to watch it with me if there's something inappropriate…or something sexual’, which she said included ‘kissing or hugging’.

Three participants spoke about their thoughts on gay and lesbian people. In response to the demographic question about sexual orientation, Angelina [heterosexual, Christian] said, ‘I would say I'm attracted to guys. Not females, because why would I? I don't want to be lesbian. Why would I be lesbian? I don't want to be lesbian’. Dennis [bisexual, Christian] had a neutral opinion about sexual minorities: ‘I don't care if people like their [own] gender, or they like something else. I mean, that's fine. 'Cause I like people’. Leah [homosexual, no religious affiliation] and Harriet [bisexual, Catholic] voiced their opinions about transgender and nonbinary people. When the interviewer asked Harriet [bisexual, Catholic] if she identified as transgender, she replied, ‘No. I kinda think that's gross’. Leah [homosexual, no religious affiliation] saw that the response options for the transgender identity question on the survey form included nonbinary, which she appreciated, saying, ‘I like that they added the nonbinary. I really like that’.

### 4.2 Major Theme 2: Religious Beliefs and Attitudes

While one student declined to respond, 14 students responded to questions about their religion and its effect (or lack thereof) on their views of sexuality or gender. Each of the three subthemes is discussed below. The first two subthemes (i.e., *Religion as Uninfluential* and *Religion as Influential*) were treated as mutually exclusive codes. The third subtheme (i.e., *Sexual Conservatism*) was not mutually exclusive, as participants could simultaneously describe religion as influential and discuss its role in shaping restrictive attitudes and beliefs (see Marshall's quote below).

#### 4.2.1 Subtheme 2.1: Religion as Uninfluential (On Sexuality/Gender Attitudes)

Five students mentioned that religion was not influential or important when learning about or forming their own views about sexuality or sexual health. Harriet [bisexual, Catholic] said that her sexuality ‘doesn't affect [her] religion’ and vice versa. Pamela [no specified sexuality, Catholic] mentioned that her religion does not influence how she views sexuality ‘because [her religion] doesn't know about [her sexuality]’. Similarly, Brian [heterosexual, Christian], Hugh [heterosexual, Christian] and Lillian [heterosexual, no religious affiliation] simply replied ‘no’ when asked if religion influenced their sexual attitudes. Two of the participants were not able to articulate any effects of religion on sexuality. When asked whether their religious views impact how they see sexuality, Isabel [no specified sexuality, Catholic] said she was ‘not sure’, and Jack [bisexual, Christian] responded, ‘I don't know’.

#### 4.2.2 Subtheme 2.2: Religion as Influential (On Sexuality/Gender Attitudes)

Seven participants indicated that religion does impact how they view sexuality, though not in a way that restricts how they act. As described above, Hugh did not think that religion was influential for him personally but explained that he could see how Christianity may affect sexuality for ‘maybe some people’. Angelina [heterosexual, Christian], Oscar [mostly heterosexual, Jewish] and Serena [heterosexual, Catholic] only said that it is influential, while the other three participants elaborated.

Earlier in the interview, Dennis [bisexual, Christian] explained that he identified as bisexual but that his religion does not approve of it. Specifically, Dennis said that his religion says that everyone should like the opposite gender, but Lucas said, ‘I guess so…like I said, I'm Christian, so I follow a lot of what the Bible says…but I don't care—I mean, if people like their gender or they like something else, I mean, that's fine’.

Marshall [heterosexual, Catholic] also expressed that religion was influential in the way he viewed sexuality, saying that he does not ‘participate in certain sexual acts because of [his] religion’. Marshall later goes on to describe how his understanding of marriage was shaped by his religious beliefs, impacting how he approached his sexuality and the decisions he made about not engaging with his sexuality until after marriage.

Leah [homosexual, no religious affiliation] likened elements of her Catholic upbringing to a “cult.” For example, she explains how, ‘When I was a kid, I was like, oh my God, this is awesome. God is awesome. And when I grew up, I'm like, that's some cult shit’. Leah later goes on to describe receiving “the talk” with her parents and at her Catholic school, which she explains ‘was ridiculous and sexist’.

#### 4.2.3 Subtheme 2.3: Sexual Conservatism

Three participants thought that their attitudes and behaviours on sexuality were shaped by religion in a restrictive manner. Brett [heterosexual, Christian] reported some dissonance over whether his Christianity affects how he views sexuality, saying:

Yes and no. I mean, obviously, God says, you know, do not, like, watch the bad stuff, like porn and stuff like that, obviously, which I tend to stay away from. But just, I don't know, you know, is‐, like even in the Bible it says don't have sex until you're married and stuff.

Brett went on to say how he is not ‘saying you cannot have sex until married, or you can't have a girlfriend and have sex’, but acknowledges that the church can hold more conservative views than some people.

Similarly, Marshall [heterosexual, Catholic] expressed that religion was influential in the way he viewed sexuality saying that he can ‘understand that there are…certain activities that go on in sexuality class that [he doesn't] agree with ‘cause of [his] religion and [his] politics’, and while he is ‘not saying that sexual activities are not okay’ but that he would want to wait until marriage to engage with his sexuality because his ‘religion says not to engage…’til marriage.’ Marshall acknowledged that his religion has influenced his restrictive beliefs surrounding sexuality but recognised that this restrictive manner need not apply to all.

While Brett [heterosexual, Christian] and Marshall [heterosexual, Catholic] seemed more agreeable to the restrictive manner of their religion, Leah [homosexual, no religious affiliation] described Catholicism in the following way:

It was very like, ‘Girls cover up your shoulders, wait until marriage, always stay pure, always stay virgin! Serve your husband!’ All of that crap. ‘You can't do this. Don't do that. Don't do this. Do this. Do that’. Going to church. Leading church. All that crap.

### 4.3 Major Theme 3: Homophobia in Religious Contexts

The same three participants who mentioned heteronormative sexual conservatism also referenced the presence of homophobia in religious contexts and teachings. As someone who identifies as a lesbian, Leah [homosexual, no religious affiliation] explained that she felt a personal conflict with the teachings of her Catholic education, where she recalled being taught that ‘homosexuality is a sin’.

Brett [heterosexual, Christian] echoed this concern when discussing the experiences of.

LGBTQ+ individuals in the church. He said:

Like, if someone's, you know, gay, or bi or lesbian or whatever, or even like pansexual or something, how do you know that they are…okay with going to church and following God and stuff if they don't know how people like God or other people around them are going to open up to them?

In response to whether religion impacts his view of sexuality, Marshall [heterosexual, Catholic] highlighted a distinction between his personal beliefs on marriage and his acceptance of same‐sex relationships:

Like, I think that marriage is between a man and a woman. And I don't have a problem with people being gay; I just think that that doesn't fit the definition of marriage. I define marriage as a clear coupling between a man and a woman, and you can have same‐sex unions and stuff like that. I just don't agree with [that] 'being defined as…[marriage].

## 5 Discussion

Curricula for sexual health education for individuals with intellectual and developmental disabilities often fall short, focusing narrowly on abstinence and neglecting essential topics like consent, relationships, and LGBTQ+ inclusivity (Travers et al. 2014). The current study examines the intersection of queerness, religion and sexual health education for college students with intellectual and developmental disabilities. It explores students' attitudes towards religion and queerness and investigates how these factors shape their experiences with sexuality and gender identity.

### 5.1 Confidence, Unfamiliarity and Desexualisation

The findings regarding confidence in knowledge about and unfamiliarity with terms regarding sexual orientation and gender identity align with other studies that show that the level of sexual health knowledge varies among young adults with intellectual and developmental disabilities (Brown and McCann 2018; Hole et al. 2022). Understanding one's sexual orientation is a vital part of sexual identity development (Diamond 2006). When youth do not have the words to explain their sexual and romantic feelings or the support of those around them in acting on those feelings (Diamond 2006), which is common among young adults with intellectual and developmental disabilities (Löfgren‐Mårtenson 2009), sexual identity development can be stunted (Diamond 2006). Of note, most of the participants who could confidently self‐identify had received prior sexual health education, and most who could not confidently self‐identify had no prior sexual health education, reflecting the necessity of explicit instruction about gender and sexuality for people with intellectual and developmental disabilities (Smith et al. 2022).

Participants expressed both internalisation and rejection of the desexualisation stereotype. While Isabel [no specified sexuality, Catholic] and Angelina [heterosexual, Christian] defaulted to getting their parents' approval to engage with their sexuality, Leah's [homosexual, no religious affiliation] sister reinforced her autonomy in decisions around sexual relationships. Those who had internalised the idea of desexualisation had limited formal sexual health education, while those who rebuffed it had learned more about sexuality and sexual health and had familial support in their sexual identity development. This difference shows the liberatory potential of sexual health education for young adults with intellectual and developmental disabilities, as consistent with the literature (Cuthbert 2017; de Wit et al. 2022). The fact that the three participants who raised this issue were all women also aligns with prior studies, which show that the desexualisation stereotype is amplified for women because of heightened concerns about sexual victimisation and pregnancy (Santos and Santos 2018).

### 5.2 Informal Education and Sentiments About Sexuality and Gender

The two examples of the effects of informal education about sexuality and gender above show how online media can affect the views of people with intellectual and developmental disabilities. Leah [homosexual, no religious affiliation] said that her belief in clothing as genderless came from spending time in online LGBTQ+ spaces, while Brett [heterosexual, Christian] learned the rape myth that women make false accusations of rape after having consensual sex from videos he had watched. Since formal instruction is often limited for people with intellectual and developmental disabilities, the information that they encounter informally, like through social media and family members, can influence their thoughts about gender, sexuality and sexual health (Löfgren‐Mårtenson et al. 2015). Additionally, many LGBTQ+ people with intellectual and developmental disabilities, like Leah, turn to online queer communities for support because their intersecting marginalisations lead to exclusion from in‐person spaces (Smith et al. 2022).

The participants held a range of views about gender and sexual minorities. This variety demonstrates that sentiments about LGBTQ+ people among college students with intellectual and developmental disabilities are not all negative or positive but diverse (Smith et al. 2022), like in the general college student population (Woodford et al. 2012). Disapproval of queerness may be more common in some disability communities because of the desire to avoid being further marginalised (Cuthbert 2017). Opportunities to change these beliefs are limited, since sexual health education programmes for people with intellectual and developmental disabilities tend to lack content about queer identity, and access to informal means of education, such as watching television and engaging with members of the LGBTQ+ community, may be restricted (Hole et al. 2022). Caregiver control over media consumption is common among people with intellectual and developmental disabilities (Löfgren‐Mårtenson et al. 2015), and the larger LGBTQ+ community is often not welcoming of queer people with intellectual and developmental disabilities in social settings (Simić Stanojević et al. 2023; Smith et al. 2022). Exposure to diverse people and perspectives led students in the current study to be more accepting of LGBTQ+ people, reinforcing the importance of queer inclusivity in sexual health education, particularly for queer students (Smith et al. 2022). How this occurs in practice, given the aforementioned challenges, remains a crucial area for future research.

### 5.3 Influence of Religion on Views of Sexuality and Gender

Religion often plays a central role in shaping an individual's values and beliefs, including those about sexuality (Lefevor et al. 2021; Santinele Martino 2022). Some religions include teachings about sexual relationships, such as sex being confined to a marital relationship, talking about sex with other people being taboo, and marriage only being between one man and one woman. Sex is less of a focus for some other religious traditions. These teachings may take place during formal educational sessions or be reinforced during informal exchanges between congregants in the religious organisation. Similar to previous research, the influence of religion on sexuality and gender attitudes among participants varied widely (Page and Shipley 2020; Santinele Martino 2022).

Five students indicated that religion had little to no impact on their personal experiences with sexuality. In contrast, seven participants acknowledged that religion influenced their views, with some aligning closely with religious teachings. For example, Marshall [heterosexual and Catholic] noted that his religion guided his decisions to refrain from sexual activity until marriage, while Lucas balanced his adherence to Christian beliefs with an acceptance of diverse sexual orientations. Two participants, Isabel [no specified sexuality and Catholic] and Jack [bisexual and Christian], were unable to articulate whether religion played a role in their sexual attitudes, suggesting that for some, the connection may be implicit, unclear, or nonexistent. These findings are consistent with previous research that suggests that the role of religion in shaping attitudes is nuanced, often varying based on personal experiences and the degree of religious engagement (Page and Shipley 2020; Santinele Martino 2022).

Some participants explicitly cited their faith as a source of restrictive attitudes towards sexuality. Brett [heterosexual and Christian] and Marshall [heterosexual and Catholic] both noted that their Christian beliefs emphasised abstinence and traditional views on marriage. Leah [homosexual and no religious affiliation], however, strongly rejected the restrictive teachings of her Catholic upbringing, criticising the emphasis on purity, gender roles and submission. Her resistance reflects a broader trend in which individuals challenge religious doctrines that conflict with personal values. These diverging findings align with studies that have examined the impact of religious conservatism on attitudes towards sexual behaviour and gender roles among people with intellectual disabilities (Lefevor et al. 2021; Santinele Martino 2022). Awareness of the diversity of religious beliefs related to sexuality among people with intellectual and developmental disabilities can help educators anticipate and address potential conflicts that may arise between these beliefs and the content presented in sexual health education programmes. This proactive approach allows for respectful dialogue, awareness and respect for diverse beliefs, and can promote feelings of inclusivity in the sexual health education classroom.

Notably, participants' sexual orientations appeared to be associated with how they interpreted and engaged with their religious beliefs. Those who identified as heterosexual were more likely to align with traditional religious teachings and emphasise abstinence or heterosexual marriage, whereas participants who identified as lesbian, bisexual, or nonreligious often expressed tension between their sexual identity and religious upbringing. This pattern suggests there could be a bidirectional relationship between sexuality and religiosity, in which lived experience with marginalised sexual identities may prompt critical reflection on restrictive religious norms or even lead to disaffiliation, as seen in Leah's rejection of her Catholic background.

### 5.4 Homophobia in Religious Contexts

Homophobia was a notable issue identified by participants, particularly in religious environments that upheld traditional definitions of marriage and condemned homosexuality. Leah [homosexual, no religious affiliation] recalled being taught that ‘homosexuality is a sin’, illustrating the alienation that many LGBTQ+ individuals face within religious settings (Lefevor et al. 2019). Brett [heterosexual and Christian] expressed concern for how LGBTQ+ individuals might struggle to feel welcomed in church communities, highlighting the tension between faith and identity. Marshall [heterosexual and Catholic], while personally accepting of same‐sex unions, maintained that marriage should be defined as a union between a man and a woman, reflecting a subtle reinforcement of exclusionary religious norms. These findings underscore the need for inclusive religious spaces and support systems for LGBTQ+ individuals, especially since research has demonstrated that religion can serve as a protective factor and support system for LGBTQ+ individuals (Lefevor et al. 2019) and individuals with intellectual and developmental disabilities (Santinele Martino 2022; Turner et al. 2004). On a college campus, student groups could fulfil this role, as queer‐inclusive groups do for LGBTQ+ students at Christian colleges (Coley 2017). Simić Stanojević et al. (2023) offer suggestions for including people with intellectual and developmental disabilities in LGBTQ+ spaces that could be applied to queer‐inclusive faith‐based student groups.

## 6 Limitations

The small sample is primarily White, cisgender, heterosexual and Christian, limiting the diversity of perspectives expressed. These participants all come from the same Midwestern college programme for students with mild‐to‐moderate intellectual and developmental disabilities; the opinions of people with more severe disabilities, who are institutionalised or otherwise unable to access a college programme for students with intellectual and developmental disabilities, and those from other regions may be different. The participants had a variety of diagnoses under the intellectual and developmental disabilities umbrella. Each diagnosis may have different effects on attitudes about religion and queerness as they relate to sexual health, but the small sample size makes it impossible to detect such differences. Additionally, participants' baseline knowledge and familiarity with the language used may have shaped their responses such that those without prior exposure to terms such as ‘sexuality’ or ‘nonbinary’ may have struggled to understand the interview questions despite clarifications from the interviewers. These differences in engagement with and interpretation of this language may reflect gaps in formal or informal sexual health education. To protect the participants' privacy, this study did not differentiate between those who were and were not under guardianship. The presence of guardianship orders can increase the acceptance of infantilizing stereotypes and impede access to sexual health education (Santinele Martino and Fudge Schormans 2018).

## 7 Conclusion

College students displayed varying levels of knowledge about gender and sexual identity, with those who had prior sexual health education demonstrating greater confidence and understanding, highlighting the importance of explicit instruction in this area, particularly for queer students, who may lack access to other means to learn about their identity. In turn, the participants' knowledge influenced how they engaged with the desexualisation stereotype, as those with limited formal education were more likely to internalise desexualisation, while exposure to supportive communities and education encouraged the rejection of such stereotypes. Religion's influence on attitudes about sexuality and gender varied widely, with some participants aligning closely with conservative teachings while others resisted restrictive norms, underscoring the need for inclusive approaches to both formal and informal sexual health education. These inclusive approaches would be co‐created with people with intellectual developmental disabilities to focus on the most important areas of education for them while explicitly addressing sexual and gender diversity, using accessible language and incorporating opportunities for skill‐building (Tarasoff 2021; Wellington et al. 2025). In addition, sexual health education curricula for people with intellectual and developmental disabilities should account for the differences identified in this study, since students will enter the classroom with a range of levels of prior formal and informal education, diverse views on sexuality and gender and varied religious beliefs that may affect how they interact with the content introduced in the course. Instructors should understand these differences and prepare both themselves and the students to encounter disagreements regarding these topics during the class to encourage respectful dialogue and a productive learning experience for all.

## Author Contributions

Drs. Hayes and Vincent, along with Ms. Wolfer, contributed to the study's conception and design. Material preparation, data collection and analysis were performed by Kristin Lurie, Rebekkah Gross and Raeshann Berry. The first draft of the manuscript was written by Kristin Lurie, Rebekkah Gross and Raeshann Berry. All authors commented on previous versions of the manuscript. All authors read and approved the final manuscript.

## Funding

The authors have nothing to report.

## Ethics Statement

This study was deemed exempt from review by the Institutional Review Board of the University of Cincinnati. The procedures used in this study align with the principles of the Declaration of Helsinki.

## Consent

Informed consent was obtained from all study participants. For participants with a legal guardian, both the participant and their guardian gave informed consent.

## Conflicts of Interest

The authors declare no conflicts of interest.

## Data Availability

Research data are not shared.

## Endnotes

^1^
Our research team includes members with and without disabilities, including an individual who identifies as a disabled researcher. We use both person‐first and identity‐first language to emphasize that language is a choice.

## References

[jar70221-bib-0001] Braun, V. , and V. Clarke . 2006. “Using Thematic Analysis in Psychology.” Qualitative Research in Psychology 3: 77–101. 10.1191/1478088706qp063oa.

[jar70221-bib-0002] Brown, M. , and E. McCann . 2018. “Sexuality Issues and the Voices of Adults With Intellectual Disabilities: A Systematic Review of the Literature.” Research in Developmental Disabilities 74: 124–138. 10.1016/j.ridd.2018.01.009. 29413427

[jar70221-bib-0004] Coley, J. S. 2017. “Reconciling Religion and LGBT Rights: Christian Universities, Theological Orientations, and LGBT Inclusion.” Social Currents 4, no. 1: 87–106. 10.1177/2329496516651639.

[jar70221-bib-0005] Crehan, E. T. , A. E. Schwartz , and E. K. Schmidt . 2023. “Who Is Delivering Sexual Health Education Content to Young Adults With Intellectual or Developmental Disability? A Survey of US‐Based School‐Based Professionals and Parents.” Sexuality and Disability 41, no. 2: 189–200. 10.1007/s11195-023-09790-2.

[jar70221-bib-0006] Cuthbert, K. 2017. “You Have to Be Normal to Be Abnormal: An Empirically Grounded Exploration of the Intersection of Asexuality and Disability.” Sociology 51, no. 2: 241–257. 10.1177/0038038515587639.

[jar70221-bib-0007] de Wit, W. , W. M. van Oorsouw , and P. J. Embregts . 2022. “Attitudes Towards Sexuality and Related Caregiver Support of People With Intellectual Disabilities: A Systematic Review on the Perspectives of People With Intellectual Disabilities.” Journal of Applied Research in Intellectual Disabilities 35, no. 1: 75–87. 10.1111/jar.12928. 34240532 PMC9290116

[jar70221-bib-0008] Diamond, L. M. 2006. “What We Got Wrong About Sexual Identity Development: Unexpected Findings From a Longitudinal Study of Young Women.” In Sexual Orientation and Mental Health: Examining Identity and Development in Lesbian, Gay, and Bisexual People, edited by A. M. Omoto and H. S. Kurtzman , 73–94. APA Press.

[jar70221-bib-0009] George, R. , and M. A. Stokes . 2018. “Sexual Orientation in Autism Spectrum Disorder.” Autism Research 11, no. 1: 133–141. 10.1002/aur.1892. 29159906

[jar70221-bib-0010] Greene, A. , I. Simić Stanojević , C. Sherwood‐Laughlin , et al. 2024. “Teachers' Perceptions of Barriers to Implementing School‐Based Sexual Health Education for Students With Intellectual and Developmental Disabilities.” American Journal of Sexuality Education 20, no. 4: 536–558. 10.1080/15546128.2024.2429432.

[jar70221-bib-0011] Harrell, E. 2021. “Crime Against People With Disabilities, 2009–2019—Statistical Tables (NCJ 301367), US Department of Justice.”

[jar70221-bib-0012] Hole, R. , L. Schnellert , and G. Cantle . 2022. “Sex: What Is the Big Deal? Exploring Individuals With Intellectual Disabilities' Experiences With Sex Education.” Qualitative Health Research 32, no. 3: 453–464. 10.1177/10497323211057090. 34923868 PMC8796054

[jar70221-bib-0013] Kirkendall, A. , H. J. Doueck , and A. Saladino . 2009. “Transitional Services for Youth With Developmental Disabilities: Living in College Dorms.” Research on Social Work Practice 19, no. 4: 434–445. 10.1177/1049731508318734.

[jar70221-bib-0014] Lee, C. E. , and J. L. Taylor . 2022. “A Review of the Benefits and Barriers to Postsecondary Education for Students With Intellectual and Developmental Disabilities.” Journal of Special Education 55, no. 4: 234–245. 10.1177/00224669211013354.

[jar70221-bib-0015] Lefevor, G. T. , A. L. Beckstead , R. L. Schow , M. Raynes , T. R. Mansfield , and C. H. Rosik . 2019. “Satisfaction and Health Within Four Sexual Identity Relationship Options.” Journal of Sex & Marital Therapy 45, no. 5: 355–369. 10.1080/0092623X.2018.1531333. 30651052

[jar70221-bib-0016] Lefevor, G. T. , C. E. Huffman , and I. P. Blaber . 2021. “Navigating Potentially Traumatic Conservative Religious Environments as a Sexual/Gender Minority.” In Violence Against LGBTQ+ Persons, edited by E. M. Lund , C. Burgess , and A. J. Johnson , 317–329. Springer.

[jar70221-bib-0017] Leonard, W. , and R. Mann . 2018. The Everyday Experiences of Lesbian, Gay, Bisexual, Transgender and Intersex (LGBTI) People Living With Disability. Vol. 111. GLHV at the Australian Research Centre in Sex, Health and Society, La Trobe University.

[jar70221-bib-0018] Löfgren‐Mårtenson, L. 2009. “The Invisibility of Young Homosexual Women and Men With Intellectual Disabilities.” Sexuality and Disability 27, no. 1: 21–26. 10.1007/s11195-008-9101-0.

[jar70221-bib-0019] Löfgren‐Mårtenson, L. , E. Sorbring , and M. Molin . 2015. “‘T@ Ngled Up in Blue’: Views of Parents and Professionals on Internet Use for Sexual Purposes Among Young People With Intellectual Disabilities.” Sexuality and Disability 33, no. 4: 533–544. 10.1007/s11195-015-9415-7.

[jar70221-bib-0020] Martinello, E. 2015. “Reviewing Risk Factors of Individuals With Intellectual Disabilities as Perpetrators of Sexually Abusive Behaviors.” Sexuality and Disability 33: 269–278. 10.1007/s11195-014-9365-5.

[jar70221-bib-0021] Miller, A. M. , E. Kismödi , J. Cottingham , and S. Gruskin . 2015. “Sexual Rights as Human Rights: A Guide to Authoritative Sources and Principles for Applying Human Rights to Sexuality and Sexual Health.” Reproductive Health Matters 23, no. 46: 16–30. 10.1016/j.rhm.2015.11.007. 26718993

[jar70221-bib-0022] Mintz, K. T. 2018. “‘My Blessed Child Does Not Need to Know About That!’: How Should Sexual Health Educators Confront the Challenge of Religious Pluralism in Working With Individuals Who Have Intellectual Disabilities?” Ethics, Medicine and Public Health 5: 8–17. 10.1016/j.jemep.2018.03.003.

[jar70221-bib-0023] Onstot, A. 2019. “Capacity to Consent: Policies and Practices That Limit Sexual Consent for People With Intellectual/Developmental Disabilities.” Sexuality and Disability 37, no. 4: 633–644. 10.1007/s11195-019-09580-9.

[jar70221-bib-0024] Page, S. , and H. Shipley . 2020. Religion and Sexualities: Theories, Themes, and Methodologies. Routledge.

[jar70221-bib-0025] Pratt, P. E. 2025. “Exploring Comprehensive Sexuality and Relationship Education for College Students With Intellectual and Developmental Disability in Inclusive Postsecondary Education Programs (Publication No. 32167650).” [Doctoral dissertation, University of Oklahoma]. ProQuest Dissertations & Theses.

[jar70221-bib-0026] Rudolph, C. E. , A. Lundin , J. W. Åhs , C. Dalman , and K. Kosidou . 2018. “Brief Report: Sexual Orientation in Individuals With Autistic Traits: Population Based Study of 47,000 Adults in Stockholm County.” Journal of Autism and Developmental Disorders 48, no. 2: 619–624. 10.1007/s10803-017-3369-9. 29086210

[jar70221-bib-0027] Santinele Martino, A. 2022. “‘I Hang Out With Non‐Christians All the Time. I Just Won't Date Them’: The Role of Religion in the Intimate Lives of Adults With Intellectual Disabilities.” Journal of Applied Research in Intellectual Disabilities 35, no. 4: 948–954. 10.1111/jar.12921. 34219330

[jar70221-bib-0028] Santinele Martino, A. , and A. Fudge Schormans . 2018. “When Good Intentions Backfire: University Research Ethics Review and the Intimate Lives of People Labeled With Intellectual Disabilities.” Forum: Qualitative Social Research 19, no. 3: 9. 10.17169/fqs-19.3.3090.

[jar70221-bib-0029] Santos, A. C. , and A. L. Santos . 2018. “Yes, We Fuck! Challenging the Misfit Sexual Body Through Disabled Women's Narratives.” Sexualities 21, no. 3: 303–318. 10.1177/1363460716688680.

[jar70221-bib-0030] Schmidt, E. K. , B. N. Hand , S. Havercamp , C. Sommerich , L. Weaver , and A. Darragh . 2021. “Sex Education Practices for People With Intellectual and Developmental Disabilities: A Qualitative Study.” American Journal of Occupational Therapy 75, no. 3: 7503180060. 10.5014/ajot.2020.044859. 34781351

[jar70221-bib-0031] Simić Stanojević, I. , M. Baugh , K. M. Greer , J. Piatt , and W. Yarber . 2023. “Increasing Opportunities for Healthy Sexual Socialization in LGBTQ+ People With IDDs: The Role of LGBTQ+ Organizations and Community.” Sexuality and Disability 41: 1–11. 10.1007/s11195-023-09789-9. PMC10127948 37362800

[jar70221-bib-0032] Smith, E. , T. M. Zirnsak , J. Power , A. Lyons , and C. Bigby . 2022. “Social Inclusion of LGBTQ and Gender Diverse Adults With Intellectual Disability in Disability Services: A Systematic Review of the Literature.” Journal of Applied Research in Intellectual Disabilities 35, no. 1: 46–59. 10.1111/jar.12925. 34309149

[jar70221-bib-0033] Tamas, D. , N. Brkic Jovanovic , M. Rajic , V. Bugarski Ignjatovic , and B. Peric Prkosovacki . 2019. “Professionals, Parents, and the General Public: Attitudes Towards the Sexuality of Persons With Intellectual Disability.” Sexuality and Disability 37: 245–258. 10.1007/s11195-018-09555-2.

[jar70221-bib-0034] Tarasoff, L. A. 2021. “A Call for Comprehensive, Disability‐and LGBTQ‐Inclusive Sexual and Reproductive Health Education.” Journal of Adolescent Health 69, no. 2: 185–186. 10.1016/j.jadohealth.2021.05.013. 34303441

[jar70221-bib-0035] Thornton, A. , and D. Camburn . 1989. “Religious Participation and Adolescent Sexual Behavior and Attitudes.” Journal of Marriage and the Family 51, no. 3: 641–653. 10.2307/352164.

[jar70221-bib-0036] Travers, J. , M. Tincani , P. S. Whitby , and E. A. Boutot . 2014. “Alignment of Sexuality Education With Self‐Determination for People With Significant Disabilities: A Review of Research and Future Directions.” Education and Training in Autism and Developmental Disabilities 49: 232–247.

[jar70221-bib-0037] Turner, S. , C. Hatton , R. Shah , J. Stansfield , and N. Rahim . 2004. “Religious Expression Amongst Adults With Intellectual Disabilities.” Journal of Applied Research in Intellectual Disabilities 17: 161–171. 10.1111/j.1468-3148.2004.00192.x.

[jar70221-bib-0038] Warrier, V. , D. M. Greenberg , E. Weir , et al. 2020. “Elevated Rates of Autism, Other Neurodevelopmental and Psychiatric Diagnoses, and Autistic Traits in Transgender and Gender‐Diverse Individuals.” Nature Communications 11, no. 1: 3959. 10.1038/s41467-020-17794-1. PMC7415151 32770077

[jar70221-bib-0039] Wellington, M. , A. Dew , P. Frawley , J. David , and A. O'Shea . 2025. “Engaging People With Intellectual Disability in Participatory Action Research: Co‐Developing Sex Educational Resources.” Sexuality and Disability 43, no. 1: 8. 10.1007/s11195-024-09886-3.

[jar70221-bib-0040] Woodford, M. R. , P. Silverschanz , E. Swank , K. S. Scherrer , and L. Raiz . 2012. “Predictors of Heterosexual College Students' Attitudes Toward LGBT People.” Journal of LGBT Youth 9, no. 4: 297–320. 10.1080/19361653.2012.716697.

